# The material stock needed to reduce disparity in access to basic services: a case study of India, across spatial scales

**DOI:** 10.1038/s42949-025-00301-0

**Published:** 2025-11-21

**Authors:** William Mihkelson, Hadi Arbabi, Stephen Hincks, Danielle Densley Tingley

**Affiliations:** 1 School of Mechanical, Aerospace, and Civil Engineering, Sir Frederick Mappin Building, Mappin Street, Sheffield, S1 3JD England; 2 School of Geography and Planning, Geography and Planning Building, Winter Street, Sheffield, S3 7ND England

**Keywords:** Climate sciences, Engineering, Environmental sciences, Environmental social sciences

## Abstract

The relationship between built environment stocks and living standards is critical to sustainable development. Yet the coupling of environmental impacts and human development outcomes warrants greater consideration. Here, we assess development outcomes associated with built environment services and quantify their relationship to the material composition of such services, across scales, for the first time, using India as a topical testbed. The multiscale model we present reveals that the provision of built environment services remains a challenge within India, with varying heterogeneity across spatial scales of intervention. This highlights the need for assessment across these scales to identify the most suitable intervention points. We show that brick and concrete stocks have grown in conjunction with development outcomes. Building on this, we estimate that upgrading inadequate housing would require between 2.2 and 5.3 Gt of material, which represents approximately 0.5% of the global carbon budget remaining to stay within 1.5° of warming.

## Introduction

The provision of basic services is integral to the achievement of minimum living standards. Construction materials accumulate within the built environment to form basic services such as housing, water and sanitation infrastructure. These materials account for a substantial portion of all primary materials extracted globally^1^, with the manufacturing of construction materials accounting for nearly 11% of energy and process-related carbon dioxide emissions^2^. Human development outcomes and environmental impacts have become tightly coupled. This is a particular challenge for nations in the Global South, given that they are predicted to experience unprecedented urbanisation in the coming decades^3,4^ while being challenged with deficits in minimum standards of living^5^. For a sustainable increase in urbanisation, there needs to be a simultaneous reduction in environmental impacts. The magnitude of the challenge globally remains largely unknown, given deficiencies in existing evidence bases on development levels and inadequate monitoring systems for associated environmental impacts.

The Sustainable Development Goals (SDGs) provide universal reference points for states to track and monitor progress as they development^6^. Across several studies, the monitoring imperative has been highlighted and championed. The SDGs and their associated indicators have been related to basic services^7^ and synergies and trade-offs within and between goals have previously been identified^8,9^. These trade-offs arise from the need to achieve many basic societal outcomes while avoiding increasing resource consumption. Existing studies have started to identify the crucial role that the built environment could play in helping to deliver SDGs^10,11^.

To date, the literature exploring the social, industrial, and anthropogenic metabolism and those aiming to monitor living standards remain largely siloed. This presents a major barrier to understanding current trends in built environment service provision and associated standards of living. Much of the socioeconomic metabolism research aimed at responding to the coupling of material use and development levels has focused on economic growth, population increase and how economic growth has driven the accumulation of material stocks in many nations^12–19^. However, recent research underpinned by socioeconomic metabolism perspectives also highlights that social wellbeing is not simply a result of rising GDP but is related to the services provided by stocks^20–23^. While the demand for services was initially identified as a key driver for stock accumulation in dynamic material flow analysis^24^, this has more recently led to the concept of the stock-flow-service nexus^21^, which seeks to better integrate the role of material stocks and their associated service into the assessment of sustainable resource management strategies. The stock-flow-service nexus acknowledges that sustainable development involves significant changes in socioeconomic metabolism in terms of the stocks and flows of energy and material and the related human, or societal activities^21^. While it is centred around the introduction of stocks and associated services into flow-centred assessments, it begins to broaden the perspectives of socioeconomic metabolism from economic growth to stock-specific services and thus acknowledges the benefits of material stocks to human wellbeing. The stock-flow-service nexus has recently extended into the conceptual framework of basic needs^25,26^ and practice-theory^27,28^. Most recently, Streeck et al.^29^ have estimated global material needs for securing decent living standards. The stock-flow-service nexus can be associated with service provision to formalise a flexible set of indicators^26^.

Broad development metrics, such as the Human Development Index^30^ and Social Progress Index^22^, have also been shown to relate to the accumulation of in-use stocks at national scales to reveal Global trends. These studies have highlighted that increases in in-use material stocks are associated with increases in standards of living and reveal that many nations of the Global South are at incipient stages of such growth. However, adopting such metrics does not clearly define the stock-service relationship in question, confounding built environment service indicators with those focused on governance and economy, eg, *expected years of schooling* as with the Human Development Index and *inclusivity* as with the Social Progress Index. Therefore, the coupling of key SDGs is not clearly elaborated. This, combined with the opportunities and challenges posed by cities in achieving SDGs globally, means there is a clear need to chart trends at sub-national scales across many nations to better understand pathways to achieving interconnected SDGs. Despite this, novel metrics monitoring such progress are rarely adopted and evaluated alongside the associated material provision, much to the detriment of the ambition of delivering against the SDGs. Further, focusing assessments at individual spatial scales may hide contextual effects associated with living standards and the consequent coupling to material use and thus fall short of adequately capturing multiscale trends, eg, which spatial delineation between cities and states makes a difference to outcomes or the relationships we find between service provision and stock accumulation? Addressing potential multiscale variations has been shown to be important for understanding inequalities in living standards and reflecting progress towards the SDGs, revealing challenges at spatially explicit scales^31^, see ‘Methods’. There remains a clear need to integrate the associated material consumption within assessments exploring inequalities in living standards. Such consideration in the routine monitoring of standards of living is crucial to deliver the Global aim of leaving no one behind, with scholars now arguing the need to capture inequalities^32^, assess interlinkages and capture heterogeneities within and between goals^31,33^.

While the SDGs have increased efforts to eradicate poverty and improve standards of living, the resource implications of achieving basic services and at what spatial scales they are needed remain poorly understood. Specifically, there exists a lack of a systematic and quantified understanding of the coupling of material stocks and living standards across different spatial scales. This is important to understand in order to anticipate the material implications of urban growth with simultaneous enhancement in access to basic services. This paper aims to contribute to these gaps in knowledge by tackling the following research questions:

1. Is there variance in the heterogeneity of access to basic services across spatial scales?
2. What is the material composition needed for access to basic services, and what are the environmental implications of increasing their provision?

Assessments of built environment material stocks and standards of living, however, must be tackled in place to address the aforementioned research gaps. As such, we use India and its data-rich census as an important testbed with its rapid population growth^34^ and urbanisation adding over 400 million urban dwellers^35^. This study provides an important first step towards understanding multi-scale trends in built environment service provision in terms of the relationship between basic needs outcomes and material stock accumulation. In this paper, we adopt a composite sustainable development index to examine minimum living standards enabled by key built environment material stocks, ie, basic needs outcomes, and evaluate the variation in perceived outcomes across scales. We then relate our index to the material composition of residential building material stocks by developing a multivariable beta regression model. In doing so, we provide a novel assessment of living standards associated with basic services and the composition of built environment material stocks across spatial scales for the first time. Finally, we use the observed relationship to estimate the material burden associated with improvements in increasing living standards.

## Results

### Heterogeneity of access to basic services across spatial scales

**Fig. 1:**
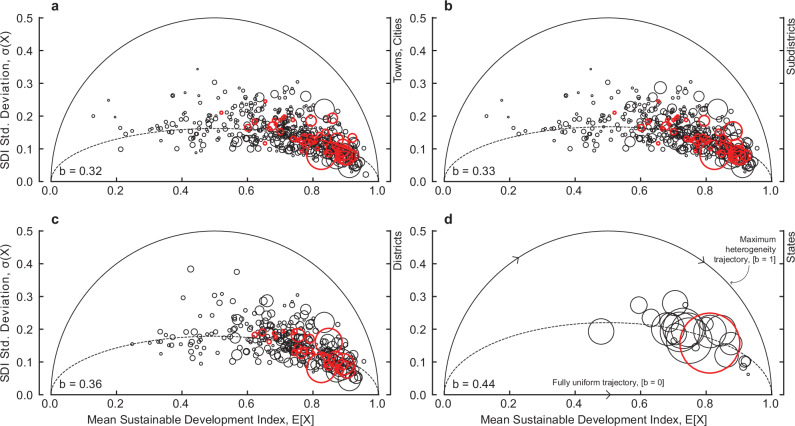
Sustainable development heterogeneity. The relationship between the standard deviation of the SDI, ${\sigma }_{i}$, and the mean SDI, ${\bar{X}}_{i}$, for different administrative scales for towns (**a**), subdistricts (**b**), districts (**c**), and states (**d**). The size of the circle is proportional to the total urban population. Red datapoints denote the largest state by population.

For India, the national level sustainable development index is found to be 0.77, indicating that on average 77% of urban households achieve access to key basic services provided by built environment stocks. This corresponds to household access rates of 84%, 79%, 62%, 71% and 93% for adequate access to housing, sanitation, treated tap water, water within the household, and electricity, respectively. However, the multiscale analysis of the sustainable development index reveals complex achievement in basic needs outcomes within and across sub-national scales, highlighting the ongoing challenge of basic service provisioning within India. At a first glance, we see a clear lag in access to services for smaller units and at smaller spatial scales, eg, towns and cities. This is illustrated in Fig. 1, where we find larger areas, ie, larger data points, tend to achieve higher mean service access with lower dispersion, ie, located towards the bottom right of Fig. 1, than smaller towns and cities. Further, the results show that smaller towns and cities tend to be located toward the centre of the profile, achieving intermediate outcomes with relatively high dispersion among neighbourhoods. However, we find significant variation in outcomes among smaller towns and cities. Taking a state with a relatively high development index as an example, highlighted in red in Fig. 1, examination of the lower spatial scales reveals the true heterogeneity of basic access to services. The results, therefore, firstly show that deficits in such outcomes tend to be lower and less varied within larger urban areas, whilst being significantly higher and more varied in smaller urban areas.

The average intra-urban heterogeneity at the scale of towns and cities, *b* = 0.32, is found such that outcomes are distributed in a way that is closer to an equal distribution of limited outcomes, ie, where *b* < 0.5 and with a variation in mean outcomes, as opposed to an all-or-nothing manner, ie, where *b* *>* 0.5. The scale dependence of these outcomes is assessed by evaluating the national basic needs profile for wards aggregated at the levels of sub-districts, districts, and states—see supplementary information for a breakdown of the geographies used. This reveals significantly larger heterogeneity among states than at lower scales, with outcomes statistically indistinguishable from each other between towns and cities, sub-districts, and districts. The multiscale analysis, therefore, finds that intra-state heterogeneity in standards of living is more significant than that in smaller spatial scales, highlighting that equitable service provision is more of a regional challenge within India than clustered among a selection of particularly populous but more deprived cities. However, the scale dependence of outcomes may be explained by overlapping definitions of many urban areas as towns or cities, sub-districts, and districts simultaneously.

**Fig. 2:**
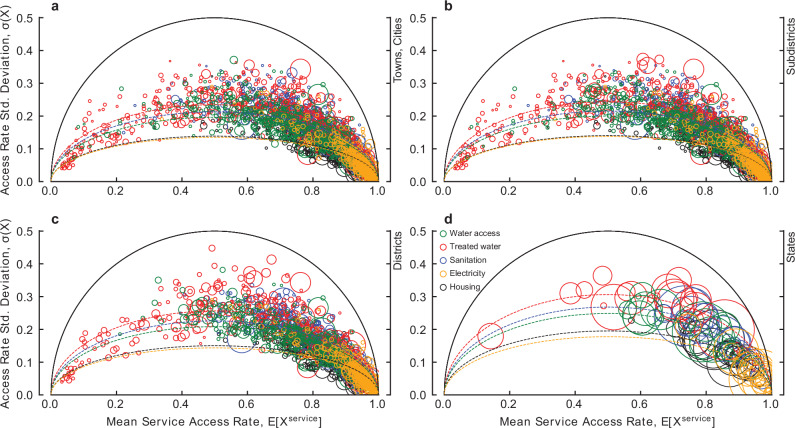
Service access heterogeneity. The relationship between the standard deviation of the household access rate ${\sigma }_{i}$ and the mean access rate, ${\bar{X}}_{i}$ for each dimension comprising the sustainable development index at each administrative scale for towns (**a**), subdistricts (**b**), districts (**c**), and states (**d**). The size of the circle is proportional to the total urban population.

Exploring access to different basic services, Fig. 2 and Table 1, the average intra-urban heterogeneity for each dimension of the sustainable development index reveals that access to water and sanitation are most challenging to basic needs. These dimensions experience the most significant intra-urban inequalities at each scale and therefore have much higher variation in access rates compared to housing and electricity access. However, the results also highlight a significant variation in outcomes across urban areas. This is particularly the case for the provision of water and sanitation infrastructure which have a significant variation in the severity of challenges between urban areas of predominantly smaller populations, ie, a range of mean access rates and dispersion of access rates among smaller data points shown in Fig. 2. The relative magnitude of heterogeneity between dimensions remains similar across scales, with inequalities in the achievement of electricity being least pronounced and with inequalities in the achievement of treated tap water access being most pronounced at each scale. The results also show that the distribution of treated tap water access is closer to an all-or-nothing case at the state level, revealing the increased magnitude of unequitable service provision at this scale.

**Table 1:** Average heterogeneity among dimensions for each scale of analysis

Scale	Dimension	Estimate, *b*	95% CI	Fit, *r*^2^
Towns/cities	SDI	0.32	[0.30, 0.35]	0.91
	Treated tap water	0.47	[0.44, 0.50]	0.93
	Sanitation	0.43	[0.40, 0.45]	0.94
	Water location	0.39	[0.37, 0.41]	0.94
	Housing	0.28	[0.27, 0.29]	0.90
	Electricity	0.27	[0.25, 0.29]	0.84
Sub-districts	SDI	0.33	[0.31, 0.36]	0.91
	Treated tap water	0.49	[0.45, 0.52]	0.93
	Sanitation	0.43	[0.41, 0.46]	0.95
	Water location	0.40	[0.38, 0.42]	0.94
	Housing	0.28	[0.27, 0.29]	0.90
	Electricity	0.28	[0.26, 0.30]	0.85
Districts	SDI	0.36	[0.33, 0.38]	0.91
	Treated tap water	0.51	[0.48, 0.54]	0.93
	Sanitation	0.45	[0.42, 0.49]	0.95
	Water location	0.42	[0.39, 0.44]	0.94
	Housing	0.30	[0.28, 0.32]	0.91
	Electricity	0.29	[0.27, 0.31]	0.86
States	SDI	0.44	[0.41, 0.47]	0.98
	Treated tap water	0.61	[0.57, 0.65]	0.99
	Sanitation	0.54	[0.51, 0.56]	0.99
	Water location	0.50	[0.47, 0.53]	0.99
	Housing	0.39	[0.35, 0.43]	0.98
	Electricity	0.36	[0.28, 0.43]	0.94

### Housing stock composition and basic access to services

**Fig. 3:**
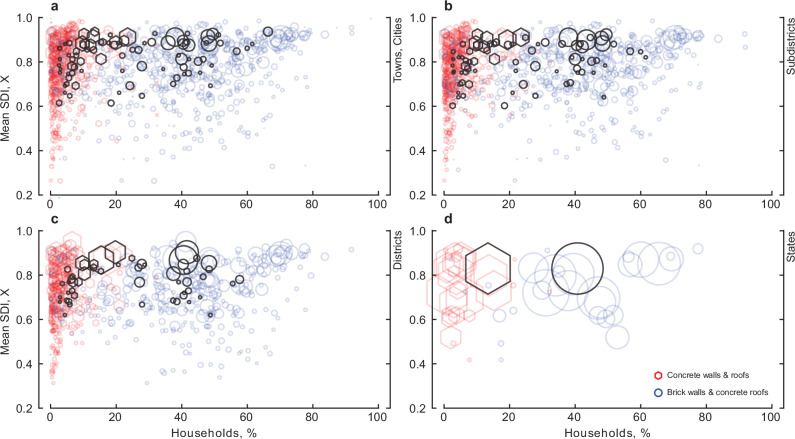
Sustainable development and construction materials. The percentage of households composed of brick walls and concrete roofs (BCHH) and concrete walls and roofs (CCHH) versus the sustainable development index with the size of the data point indicating the absolute number of households within the urban area by each administrative scale for towns (**a**), subdistricts (**b**), districts (**c**), and states (**d**). Black datapoints denote the largest state by population.

Uplift in basic access to services is often a result of or co-occurring with urban population growth and further urbanisation, Fig. 1. Here, we now focus on exploring the composition of housing stock material as a proxy signature of the material implications of increased access to services. Unsurprisingly, the results reveal that the prevalence of brick wall and concrete roof households (BCHH) and concrete wall and concrete roof households (CCHH) is related to overall basic needs outcomes, see Table 2 and Fig. 3. Table 2 estimates show that increases in the composition of such households are associated with increases in basic needs outcomes. Univariate analysis reveals the opposite relationship for less common household compositions, such as those using mud for walls and metal sheets for roofs. However, these compositions are much less prevalent across the towns and cities of India, see the ‘Methods’ section, and therefore do not describe a significant composition of the overall built environment material stocks. A greater composition of brick and concrete material in the built environment stock coincides with greater achievement of overall basic needs outcomes. Specifically, a 1% increase in the composition of BCHH is associated with an average 0.2% increase in the sustainable development index, and a 1% increase in CCHH is associated with an average 0.5% increase in the sustainable development index. Urban areas containing a greater composition of brick and concrete within built environment stocks tend to have, albeit marginally, higher overall basic needs outcomes. Due to the composition of households, the results also suggest that the prevalence of concrete stocks has grown in conjunction with overall basic needs outcomes to a greater extent than for brick stocks. The derivatives are statistically indistinguishable from each other across scales, except at the largest spatial scales, where we see no coupling at the state-level between the increased prevalence of CCHH and overall basic needs outcomes.

**Table 2:** Services access and material composition

Scale of analysis	Dimension	Household composition	Average marginal effect
Towns/cities	Housing	BCHH	0.0012
		CCHH	N/Aa
	Sanitation	BCHH	0.0013
		CCHH	0.0033
	Treated tap water	BCHH	0.0017
		CCHH	0.011
	Water location	BCHH	0.0016
		CCHH	N/A
	Electricity	BCHH	0.00053
		CCHH	0.0024
Subdistricts	Housing	BCHH	0.00088
		CCHH	N/A
	Sanitation	BCHH	0.0010
		CCHH	0.0047
	Treated tap water	BCHH	0.0012
		CCHH	0.013
	Water location	BCHH	0.0013
		CCHH	N/A
	Electricity	BCHH	0.00056
		CCHH	0.0033
Districts	Housing	BCHH	0.00073
		CCHH	N/A
	Sanitation	BCHH	0.0010
		CCHH	0.0047
	Treated tap water	BCHH	0.0012
		CCHH	0.015
	Water location	BCHH	0.00095
		CCHH	N/A
	Electricity	BCHH	0.00088
		CCHH	0.0053
States	Housing	BCHH	0.0044
		CCHH	N/A
	Sanitation	BCHH	N/A
		CCHH	N/A
	Treated tap water	BCHH	0.0048
		CCHH	N/A
	Water location	BCHH	0.0028
		CCHH	N/A
	Electricity	BCHH	0.00089
		CCHH	0.0025

Results of the service-specific beta regression across scales of analysis show only the variables with a significant and positive impact on overall basic needs. Note: BCHH refers to brick wall and concrete roof household compositions, and CCHH refers to concrete wall and concrete roof household compositions.

^a^Note that N/A values are shown for variables which are not found to be statistically significant, ie, *p*-value » 0.05.

We now further explore the relationship between the decomposed sustainable development index, ie, individually examining access to housing, sanitation, treated tap water, water location and electricity, in relation to BCHH and CCHH, Table 2. While we see similar patterns for BCCH and individual services as before, CCHH shows a more varied co-occurrence with individual basic services. Increasing CCHH is not associated with outcomes of housing or adequate, on-premise, water access. CCHH is also only associated with improved access to electricity at the state level, exhibiting an average 0.3% increase in the sustainable development index for a 1% increase in its prevalence.

### Material requirements for uplifting basic needs outcomes

Given that the prevalence of brick and concrete stocks is linked to higher overall basic needs outcomes, we now explore the implications of upgrading existing housing to consistent brick/concrete stock type after Mihkelson et al.^36^. We explore three scenarios successively escalating in scale in their definition of inadequate housing to be replaced as outlined in Table 3.

**Table 3:** Inadequate housing

Scenario	Definition of inadequate housing	Notes
**1**	Inadequate housing is defined as households which are not permanent.	Definition used to define inadequate housing^31,47,51^.
**2**	Inadequate housing is defined as those households which are not constructed from: 1) Brick walls and concrete roofs, or 2) Concrete walls and concrete roofs.	Based on the observation that a greater composition of such households across urban areas is associated with greater basic needs outcomes.
**3**	Inadequate housing is defined as households which are not constructed from brick walls or concrete roofs.	

Definitions used to calculate the deficits in housing provision across three different scenarios. Note that all scenarios are compared at the nationally aggregated scale to facilitate comparison to existing literature; however, only scenario 1 is related to changes in basic needs outcomes sub-nationally.

We use Mihkelson et al.^36^ material stocks intensity from the Indian city of Chandigarh as an example of a quickly developed master planned city, which aimed to provide high standards of living. This work estimated material stocks at approximately 216 tons/household, equivalent to 370 GJ/household or 52 tonsCO_2_e/household.

**Fig. 4:**
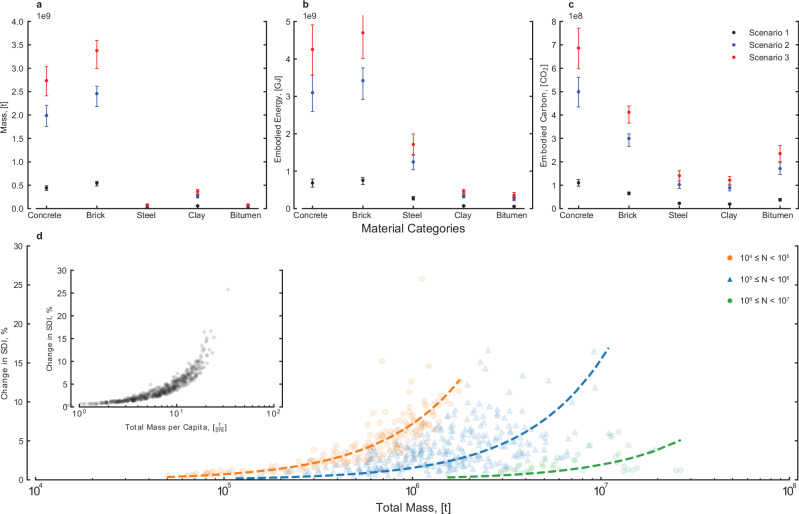
Change in SDI versus construction material needed. National material (**a**), embodied energy (**b**), and embodied carbon (**c**) requirements for each scenario. Note that error bars reflect material stock variations from ref. ^36^. Total material stock required versus percentage increase in the SDI, ${X}_{i,\mathrm{improved}}$ (**d**) with *N* depicting urban unit population range. Panel **d** inset shows the required material stocks per capita versus percentage increase in SDI, ${X}_{i,\mathrm{improved}}$.

Figure 4a–c illustrates our findings for each of the three scenarios. For scenario 1, it is estimated that a little over 10 million households are classified as inadequate, requiring approximately 2.2 Gt of material to ensure universally adequate housing. The equivalent EC and EE are approximately 0.54 GtCO_2_e and 3.8EJ, respectively. In scenario 2, it is estimated that 21.1 million households are inadequate. Homogenising the total Indian housing stock to that of Chandigarh, in Scenario 3, results in approximately 24.5 million households requiring upgrades.

At a national level, the sustainable development index increases by only 0.02 from 0.79 following the largest upgrade scheme in scenario 3. Housing provision as a proxy for increased services access requires a substantial quantity of material for minimal uplift in overall basic needs outcomes. At lower spatial scales, however, the picture is more nuanced. Figure 4d shows the total mass required for the corresponding uplift in basic needs outcomes by urban size. We see how larger urban areas require a significantly larger provision of material stock to upgrade inadequate housing for relatively low improvements to overall basic needs outcomes compared to smaller urban areas. Smaller urban areas generally require significantly lower total material stock, but much larger material stocks per capita for a higher marginal improvement to overall outcomes.

## Discussion

There are policy implications of what is considered a ‘place’. Assessments of trends concerning the provision of material stocks and development are often focused on individual units at specific scales of analysis within nations, such as prefectures or cities^13,37^, with those concerning standards of living focused on the Global scale^22,29,30^. Multiscale analysis of place is, however, important given different governing bodies operating at or across these levels, some of which may be better equipped to coordinate intervention efforts from both a material and development perspective.

We have shown that disparity in access to basic services is more acutely observable at smaller urban scales. Single-scale considerations overlook significant deprivation in many smaller towns and urban areas and mask these disparities at the regional and state levels. Focusing on deprived and smaller towns and cities enables larger- or state-level master plans to run through spatial scales. Yet, addressing these without incurring significant material costs would still require instilling material efficiency strategies early in the development of urban areas^36^ to ensure that lock-in effects are avoided and circular economy strategies are introduced early to limit primary material consumption in future urban development.

Meeting material demand of decent living standards will mean doing more with less. Upgrades to inadequate housing are assumed to be net additions to stocks, ie, through the provision of new housing units with or without demolition of existing stocks. There may however be opportunities to upgrade existing housing, or reuse elements from this, which would reduce new resource consumption, however, quantification of this reuse potential is outside the scope of this study. This assumption of net additions to stock has implications for the per capita in-use stock of residential buildings within India. For example, the net additions to stock in scenario 1, are in the range of 6.2–7.6 tons/capita nationally, with an average of 7.0 tons/capita based on the uncertainty in values for material stocks required per household. This is equivalent to approximately 30% of China’s per capita in-use stock of residential buildings in 2008^38^. It is also important to note that per capita levels of material stocks vary significantly between countries. Streek et al.^29^ show an average stock of 140t/cap in high income countries compared to an average of 21t/cap in low income countries.

The required material stocks per capita, however, may reduce based on the way in which housing is provided. For example, higher-rise construction than that of Chandigarh could result in a lower material intensity, kg/m^2^, as shown in Rio de Janeiro^39^. This can also lead to additional efficiencies from agglomeration effects. Material savings could be further compounded by integrating minimum floor area provision^40,41^, meaning that the floor area provision assumed on average per household would reduce, with a corresponding reduction in material stocks to upgrade all housing. Higher-rise construction, combined with lower floor area per household implies increasing densification of urban areas. This is particularly important given the unprecedented rates of urbanisation and population growth expected in coming decades^42,43^, as well as the significant increase to housing and urban infrastructure provision expected in the coming years^44^.

Reducing per capita floor area, however, may be in tension with the pursuit of economic growth, which is generally considered a main driver for floor space growth^45^, and is part of India’s ‘inclusive growth’ strategy which outlines the goals of increased GDP and reductions to urban poverty in terms of basic service access^46^. On the other hand, upgrading housing does also present an opportunity to improve the energy efficiency of the building stock, and thus reduce operational carbon emissions of the stock. This upgrade has been the subject of a number of studies which have explored implementation of varying degrees of energy-saving strategies to further reduce energy requirements^41,47,48^.

Finally, global trends indicate that in-use stock provision may increase significantly in both developed and developing countries, despite many nations of the Global North already achieving near universal access to basic services^29^. As such, many sustainable development goals related to basic service access are achieved in the Global North at a historically high carbon cost. This has restricted the available emissions budget of resources for less developed countries to achieve a minimum living standard.

Achieving the SDGs is a Global Agenda and a Global responsibility with a clear need for consensus on what constitutes a “minimum standard of living”. There is, therefore, a clear need to better integrate assessments of basic human needs into such indicators, such as those measuring stock productivity in recent stock-flow-service nexus literature^26^, to better capture trade-offs between indicators. For example, adopting a material stocks per service, or material stocks per capita per service, indicator may ensure that the reporting of the material requirements to ensure minimum living standards integrates the needs of other sustainable development goals. This may adequately identify the stock-flow-service nexus in question to capture trade-offs between sustainable development goals and emissions and better inform strategies for decoupling.

We have estimated India to be expected to require between 0.5 and 1.3 GtCO_2_e to upgrade all its inadequate housing. This is up to 0.5% of the total remaining Global carbon budget for limiting global warming to 1.5 degrees Celsius^49^. Although small as a fraction, the potential emissions associated with the housing upgrade would equal the *annual* global carbon emissions reduction required. If India builds in resource efficiency strategies, this may reduce both the upfront and future resource requirements. This work, however, has specifically explored the material associated with upgrading housing, where housing upgrade has been used a proxy for widespread infrastructure development. Other studies have also highlighted the significantly lower material requirements for waste water and water supply pipes^50^ as well as the energy requirements associated with the operation of such services within India^51^. Thus, significantly greater improvements to overall basic needs outcomes are expected to be possible for lower per capita material requirements.

## Methods

The overarching approach here is to firstly quantify standards of living and the associated heterogeneity at the scales of towns and cities, sub-districts, districts, and states. To achieve this, data provided through the Census of India for 2011^52^ measuring household access to amenities and assets is formalised within an average measure of basic needs outcomes. This refers to the average achievement of household access to various basic services. While the number of dimensions of basic needs varies between studies, access to basic services, namely: housing, sanitation, water and electricity, has been a common measure of basic needs in the Global South^31,53–56^ all of which relate to key built environment material stocks. Built environment material stocks are defined as stationary stocks of material within the built environment such as buildings and infrastructure and broadly relate to residential and non-residential buildings, as well as transport, communication, and energy infrastructure. Such basic service provisioning is also considered by the UN as essential for expanding basic capabilities^43^ and is indicated by SDG 1.4.1 which measures the proportion of the population with access to basic services. The measurement of such basic service access is therefore a common approach to assess basic needs outcomes in the Global South and clearly defines the stock-service relationship in question, thus offering insight into progress towards key SDGs.

From here we relate the standards of living to the composition of built environment material stocks across the same scales of analysis by formulating a multiple variable beta regression model, going beyond the limitations of standard linear regression to more accurately quantify the relationship. To achieve this, data available from the census of India relating to the number of households constructed by their predominant materials is adopted. Residential building material stocks are shown to comprise a significant share of overall built environment material stocks in developed economies such as Japan^57^, the United Kingdom^58^ and Denmark^50^. Further, a significant demand for new buildings is expected in the coming decades^59^, with over 400 million new urban dwellers expected within India to 2050^4^. As such, basic needs outcomes are related to the prevalence of certain compositions of residential building material stocks, which is used as an indicator for the overall composition of non-mobile built environment material stocks.

### Monitoring standards of living

Capabilities are at the centre of human development^60^ and have shifted the focus from measures of income and consumption toward approaches that attempt to measure the ways in which households reside and work within their environment^60,61^. The United Nations now acknowledge the need for member states to create context-specific indicators that go beyond national averages and that complement the global indicator framework sub-nationally^43,62^. Various indices have emerged assessing human development, with many studies adopting index- and indicator-orientated frameworks to measure the sustainability of cities^63^. The combination of individual indicators into a single metric, or composite index, is a common approach when adopting indicators to monitor development outcomes^64^. Studies have adopted the well-established methodological approach of measuring human outcomes by assessing the average achievement of indicators in an area^31^, an approach typified by the Human Development Index most frequently used to measure and track national trends in human development^65,66^.

A central challenge to the monitoring of development outcomes is tied to heterogeneity due to the outcomes among a population being more varied than when evaluated by simple averages^67^. The presence of inequalities may impact the representativeness of average measures such that policy may become regressive and have unintended consequences for those furthest behind as highlighted in the distributional effects literature^67–70^. This may be exacerbated by the fact that city growth is generally associated with increased inequality in access to urban infrastructure^71,72^. Such consideration in the routine monitoring of standards of living is crucial to deliver the Global Agenda’s aim of leaving no-one behind. As such, scholars now argue the need to capture inequalities^32^, assess interlinkages and capture heterogeneities within and between goals^31,33^. These recommendations point to the need to develop alternative and flexible approaches to ensure the efficacy of indicators as a policy instrument^64^. A key study addressing such challenges and monitoring basic service access in the context of the SDGs has developed a multiscale model using an average composite index, with the aim to understand urbanisation in terms of average outcomes and associated inequalities^31^. The study presents an approach to capture potential variation in outcomes across spatial scales, capturing challenges that may be experienced at spatially explicit scales. This is an important consideration in the assessment of sustainable development as the choice of urban unit, or the scale to which we aggregate, may have implications for urban policy and thus the achievement of the SDGs.

### Quantifying basic needs profiles

We adopt the census data^52^ to formulate a sustainable development index (SDI)^31^ measuring the average household outcomes of basic needs relating to water, sanitation, housing, and electricity. The areal household access data is bound between 0 and 1 indicating a range of access in respective dimensions from 0% to 100%, respectively. The dimensions are then aggregated via a geometric mean, Eq. 1, to create the final index for all wards of towns and cities containing over 30 wards, see Table 4 for summary data.

**Table 4:** Summary data of household counts for wards, towns/cities, sub-districts, districts, and states

Statistics	Wards	Towns/cities	Sub-districts	Districts	States
No. of data points	23,192	524	486	321	24
Maximum	156,619	2,101,831	2,101,831	2,101,831	8,684,761
Minimum	1	6289	6289	8518	17,807
Mean	1759	77,853	83,941	127,087	1,699,798
Std. dev.	3614	176,993	189,193	245,757	2,004,313

Equation 1 shows the aggregation of $n$ dimensions, for area $i$, to calculate the SDI, ${X}_{i}$. No weighting is used for the sustainable development index and therefore the overall index is a measure of the non-weighted average achievement of the normalised dimensions^73^. The geometric mean is often adopted to capture outcomes such that the emphasis is on the achievement of all dimensions, implying that they are not substitutional, with no weighting adopted to avoid the normative judgement of the relative importance of each dimension^56^. This is fitting with the notion that universal access to basic services is required as part of achieving the various SDGs and for providing a minimum standard of living. We therefore formalise the sustainable development index as in Eq. 2, with dimensions listed in Table 5 and with the adopted definitions of achievement being coherent with the literature addressing minimum standards of living^31,74,75^ as well as broader monitoring imperatives such as the Multidimensional Poverty Index^76^ and SDGs.

1 $${X}_{i}=\root{{n}}\of{{\prod }_{j=1}^{n}{X}_{i}^{j}}$$

2 $${X}_{i}=\root{{5}}\of{{X}_{i}^{\mathrm{water}}{X}_{i}^{\mathrm{water}\,\mathrm{location}}{X}_{i}^{\mathrm{housing}}{X}_{i}^{\mathrm{sanitation}}{X}_{i}^{\mathrm{electricity}}}$$

**Table 5:** Sustainable development index (SDI) dimensions

Dimension	Definition	SDG reference	
Sanitation	Flush/pour latrine (piped sewer system, septic tank or other) or pit latrine with slab/ventilated improvement	SDG 6.2.1	SDG 1.4.1
Main source of drinking water	Tap water from a treated source	SDG 6.1.1	
Availability of drinking water source^a^	Drinking water is found within the premises	SDG 6.1.1	
Housing	Permanent housing	SDG 11.1.1	
Electricity	Electricity used for lighting	SDG 7.1.1	

^a^Availability of drinking water source is combined with the main source of drinking water indicator in previous studies in Brazil and South Africa due to the aggregation of each indicator in the respective census datasets (Brelsford et al. ^31^). The Census of India records this indicator separately and is therefore included explicitly within the SDI. Appropriate water dimensions are identified for India through Spearman and Pearson correlation.

These measure the average outcomes of basic service access using census data for the year 2011. The definitions broadly relate to those from analyses in South Africa and Brazil and are related to their respective SDGs and indicators based on their definition and identified interconnectivity presented by the UN.

We then explore the national basic needs profile by evaluating the average intra-urban heterogeneity in the sustainable development index across administrative scales. To assess intra-urban heterogeneity and multiscale effects, we follow a common methodology outlined in previous studies assessing heterogeneity of basic needs outcomes in South Africa and Brazil, which measure the relative levels of spatial heterogeneity in basic service access between regions^31^. We therefore aggregate wards, ie, the local urban areas of India, to their respective towns and cities, sub-districts, districts, and states. As discussed previously, we include only those towns and cities containing 30 wards, which is adopted as a rule of thumb to increase the confidence interval of the dataset when assessing the mean and standard deviation of basic needs outcomes.

The sustainable development index is bound between 0 and 1 such that in cases where households in area, $i$, have universal access to services, ${X}_{i}$ = 1, and where all households in area, $i$, have no access to services, ${X}_{i}$ = 0. The variance of $X$ is therefore typically maximum where the mean SDI, ${\bar{X}}_{i}$, is equal to 0.5 owing to a greater number of possible variations in access within area, $i$, leading to a greater possible dispersion from the mean, ie, when half of the data points have access to services, ${X}_{i}$ = 1, and the other half have access to services, ${X}_{i}$ = 0. We model basic needs outcomes as a Bernoulli process where outcomes are modelled within area $i$ as either “success” where the area is considered as developed, or “failure” where the area is considered as not developed. The probability of success, $p$, is given where ${\bar{X}}_{i}=1$ and the probability of failure, $1-p$, is given by ${\bar{X}}_{i}=0$. We can therefore parameterise the standard deviation of ${X}_{i}$, ${\sigma }_{i}$ as:

3 $${\sigma }_{i}={b}_{i}\sqrt{{\bar{X}}_{i}(1-{\bar{X}}_{i})}$$

where the square root corresponds to the standard deviation of a random Bernoulli. As a result, the maximum and minimum variance for each value of $\bar{X}$ is $b=1$ and $b=0$, respectively. The properties of the standard deviation dictate that $b\ge 0$, and therefore profiles of outcomes are characterised by ${b}_{i}$ given ${\bar{X}}_{i}$. Figure 1 illustrates how basic needs profiles in the space of (${\bar{X}}_{i},{\sigma }_{i})$ are formed and relate to the heterogeneity index, ${b}_{i}$. We calculate the average heterogeneity index, $b$, for each scale by regressing ${\sigma }_{i}$ on $\sqrt{{\bar{X}}_{i}-{{\bar{X}}_{i}}^{2}}$, which is used to characterise profiles as a function of space and time as outcomes change in each unit $i$ and the values ${(\bar{X}}_{i},{\sigma }_{i})$ tend to (1,0). Given that we use cross-sectional data here, we consider the dimension of space indicated by the administrative regions given within the census data and limit the analysis to the year 2011. We also conduct this analysis for the decomposed sustainable development index and therefore evaluate the heterogeneity in dimensions that comprise overall basic needs. The basic needs profile exhibits behaviour typical of a Kuznets curve (179) where the profile of maximum heterogeneity, $b=1$, peaks where ${\bar{X}}_{i}=0.5$, and reaches maximum and minimum where $({\bar{X}}_{i},{\sigma }_{i})$ is (1,0) and (0,0), respectively.

### Quantifying the relationship between standards of living and the provision of built environment material stocks

The basic needs outcomes quantified by the sustainable development index are then related to the composition of residential buildings for each scale. This was achieved by developing a multivariable beta regression model. Regression models have been used in socioeconomic metabolism research relating development metrics to in-use stocks^30,77^, as well as for access to services such as sanitation in relation to per capita energy use^78^ and for consumption-based emissions with socioeconomic factors such as income and population growth^79^. Here, the dependent variable, ie, the SDI, is a measure of the rates of prevalence and is therefore bound within the range [0,1]. Therefore, due to the model specification of standard regression models, regression coefficients may yield fitted values which lie outside the upper and lower bounds of the SDI, ie, DI > 1 or SDI < 0. Although such analysis still enables an indication of the direction of the perceived relationship, ie, positive or negative impacts, the magnitude of the effects may be highly inaccurate due to unrealistic predictions. Further, proportional data like that of the sustainable development index is often distributed in an asymmetrical manner such that they may display heteroskedastic behaviour and thus yield standard linear regression inappropriate^80^. As a key objective here is to *quantify* the relationship between the prevalence of built environment material stock compositions and basic needs outcomes, it is appropriate to turn to fractional response models, which concern outcomes bound within the range [0,1]. Such models assume values within the bounded range and therefore do not concern values equal to 0 or 1. This excludes only one small town containing 69 households from the analysis, given that it achieves a sustainable development index = 0. Specifically, we develop a beta regression model, which is a relatively new fractional response model proposed by Ferrari and Cribari-Neto^81^, see Supplementary Material for more information.

**Fig. 5:**
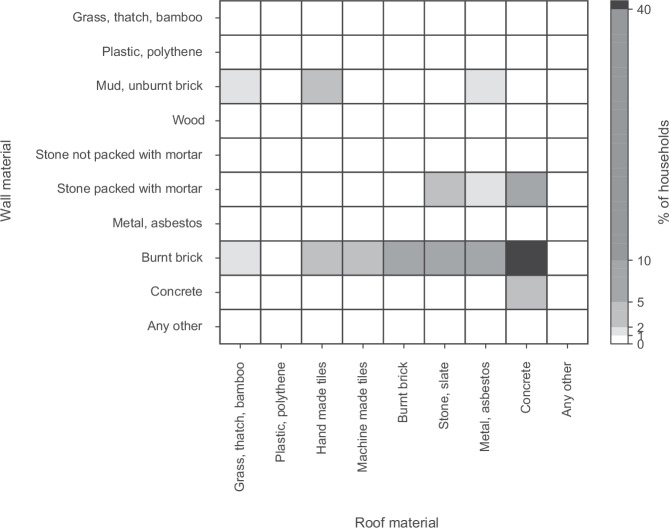
Household material composition. The average prevalence of residential buildings comprised of a unique combination of wall and roof materials for the towns and cities of India.

The census of India records the total number of households constructed of a particular combination of walls and roofs^52^. The dataset contains 90 variables, combining ten material types for walls with nine material types for roofs. However, often too many variables are included in instances where many variables are available, resulting in various limitations such as overfitting. We first process the data from counts into proportions. A key characteristic of this is that increases in the prevalence of one variable would generally lead to reductions in others. This means that the composition of built environment material stocks can therefore be more readily assessed without overfitting the model. Further, the study concerns the composition of built environment material stocks and therefore includes a significant number of variables that are rarely prevalent within the model selection itself, which may result in inaccurate variable selection. For the reasons regarding model overfitting and the proportionality of the dataset previously discussed, the number of covariates is reduced based on the average prevalence and by considering only those with a positive relationship with overall basic needs outcomes, ie, variables with a positive regression coefficient. Univariate beta regression is first performed on those variables accounting for at least 1% of the composition of households on average among towns and cities, see Fig. 5. These variables account for 14 out of the 90 available variables corresponding to over 86% of the total households within the urban areas of India. The forward selection process is then performed for variables with a positive and significant impact on basic needs outcomes. This involves sequentially including variables with the greatest impact on basic needs outcomes identified within the univariate model and including only those with a positive and significant impact in the multivariable regression. This results in two key variables that are related to improved basic needs outcomes.

### Methodological constraints

The results highlight that heterogeneity within Indian towns and cities, *b* = 0.32, is significantly lower than that in Brazil, *b* = 0.58, and South Africa, *b* = 0.57^31^ indicated by the average heterogeneity index. Despite this, the scale dependence of such challenges is consistent with these nations, with larger administrative scales exhibiting greater intra-urban heterogeneity in basic service access. However, while heterogeneity indices specific to each dimension of the sustainable development index are not calculated for Brazil and South Africa, there are key differences between all three nations, highlighting context-specific implications for built environment material stocks provision. For example, deficits in permanent housing and sanitation are found to be most significant in Brazil and South Africa, respectively, whereas deficits in access to water infrastructure are most challenging in India. There also seems to be significantly higher heterogeneity in the provisioning of basic services in these nations, whereas India has a more even distribution of, albeit limited, access to respective services. Future work should seek to assess the relationship between the existing standards of living and the composition of built environment material stocks in Brazil and South Africa to elaborate on the materiality of this development and the associated challenges for interconnected sustainable development goals. However, it is also important to consider that the methodological approach relies on a single average measure and thus contains inherent ecological fallacies, such that inter- and intra-urban challenges may be overlooked, resulting in regressive policy. Scholars now recommend the monitoring of outcomes using complementary metrics^82^, which may enable such assessments to go beyond inherent ecological fallacies incurred due to the reliance of average measures on areal data such as censuses. This points to the potential value of complementing such assessments with multidimensional poverty measures which capture the joint distribution of dimensions and thus capture those in extreme poverty^76,83^. This would aid in targeted policy making and thus complement multiscale analyses when coordinated within the planning levels of nations discussed previously. The importance of this is also emphasised, given that the Global Agenda is committed to ensuring no one is left behind and thus highlights the role of multidimensional metrics in stock-flow-service nexus assessments to ensure equitable resource provisioning.

While the presented study is unable to quantify causation, ie, whether the material composition of services directly causes improvements to standards of living, studies have suggested that this is the case. A study assessing the impacts of a large-scale housing programme in Mexico has shown that replacing mud floors with cement improves child and adult welfare and thus directly impacts basic needs^84^. While the regression model does not capture the material used within floors, BCHH and CCHH are shown to be constructed with concrete floors across various regions of India^85–88^. Further, such residential building compositions are argued as necessary for adequate housing provision among numerous studies^47,48,51,75,89^. Therefore, the current trends suggest that the prevalence of BCHH and CCHH housing directly impacts basic needs outcomes. This further underlines the importance of this coupling within the Global context as discussed earlier, reinforcing the need to understand resource efficiency strategies in the context of providing minimum standards of living. Future work should therefore seek to understand and quantify the impact of potential decoupling strategies, such as those identified within the decent living standards literature, aiming to ensure minimum service provisioning^41,75,90^, ie, only providing what is required. Combining such analysis within India as new census data becomes available in the coming years will offer greater resolution as to the development of India over the decade, as well as providing a quantification of the magnitude of decoupling and consequent sustainable development trajectory based on such strategies. It is also important to verify whether the trends observed here exist in other contexts, particularly in areas with high deficits in basic service provision. For example, in Peru where urban areas are found to have low basic needs outcomes with particularly high deficits in access to water infrastructure^91^ and with a significant proportion of brick masonry residential buildings^92–94^ as shown in the present study for many urban areas of India.

Significant challenges exist surrounding the data availability of built environment material stocks within India, limiting the integration of material flow and stock analysis within such assessments. Future work assessing characteristics of material stock accumulation within the urban areas of India is crucial to understand the relative scale of challenges associated with achieving a minimum standard of living. This would also offer an improved understanding as to the current implications for monitoring SDGs, eg, by identifying improved material indicators to capture such trade-offs, and the appropriate pathways to ensure that interconnected SDGs can be achieved simultaneously. The observed trends found here also underline the importance of such studies when considering the provision of water and sanitation infrastructure. Future work may also seek to develop population weighted beta regression approaches such that the extent to which brick and concrete material stocks is provided across urban areas is better captured. This is important because the results indicate that larger urban areas tend to have a relatively high composition of brick and concrete material stocks as well as high basic needs outcomes compared to smaller areas. Additionally, we do not evaluate the spatial distribution of variables here which may reveal spatially dependent outcomes and patterns associated with higher material stocks compositions and basic needs outcomes. Given that India spans multiple climatic and seismic zones, resulting in various material and energy requirements for residential building material stocks^47,86^, eg, through the increased requirement for steel reinforcing in seismic areas, future work should seek to verify the spatial distribution of material stocks sub-nationally to elaborate potential regional constraints associated with basic needs outcomes.

## Supplementary information

Supplementary information

## Data Availability

The underlying data used is publicly available on http://censusindia.gov.in/. The cleaned minimal dataset that would be necessary to interpret and replicate the results and figures in this paper is available publicly on https://github.com/ci1hea/material-stock-india.

## Code availability

The data and code used to generate the results and figures in this paper are available publicly on https://github.com/ci1hea/material-stock-india.
