# Supplementary material for: The material stock needed to reduce disparity in access to basic services: a case study of India, across spatial scales
Source: NPJ Urban Sustain. 2025 Nov 21;5(1):112. doi: 10.1038/s42949-025-00301-0 (PMC12722180; doi:10.1038/s42949-025-00301-0)
Supplement: Supplementary file 1 — Supplementary information [file 42949_2025_301_MOESM1_ESM.docx]

# Supplementary Information for Material Stock to Reduce Disparity in Access to Basic Services

William Mihkelson^1^, Hadi Arbabi^1^, Stephen Hincks^2^, Danielle Densley Tingley^1,*^

^1^ School of Mechanical, Aerospace, and Civil Engineering, Sir Frederick Mappin Building, Mappin Street, Sheffield, England, S1 3JD
^2^ School of Geography and Planning, Geography and Planning Building, Winter Street, Sheffield, S3 7ND

* corresponding authors

# Scales of analysis

The Government of India (GoI) outlines the multiple sector interventions at different administrative scales in their recent Reforms in Urban Planning Capacity (National Institution for Transforming India, 2021). Various responsibilities reside at different administrative scales, generally ranging from the design of policy frameworks at the national and state level down to specific urban planning projects, such as master plans and urban redevelopment plans at the local administrative scale, see Table SI1. These multiple sector planning responsibilities broadly range from national or state level responsibilities for envisioning the future development of the country which precipitates down through administrative scales, to the detailed planning of urban interventions at the local urban area, ie, towns and cities.

**Supplementary Table 1:** **Scales of analysis.** Examples of responsibilities of levels of planning across administrative scales as outlined by the Government of India.

| Planning level/administrative scale | Example of responsibilities |
| --- | --- |
| Town and city level/Municipal Corporations | Land use planning such as development plans, master plans, building construction permits and redevelopment of inner-city areas.  Mobility planning such as planning of bus/rail transit systems. Environmental infrastructure planning such as water supply and sanitation infrastructure plans. |
| Regional level/districts | District development plans, planning of highways and transportation.  Regional infrastructure such as the planning of highways and transportation infrastructure. |
| National/state level | Policy framework such as National Urban Transport Policy and National Housing and Habitat Policy.  Design of programs such as the rejuvenation of urban areas and the Smart Cities Mission as well as strategic and project planning. |

The census of India provides comprehensive coverage of access rates to basic services across urban India for each administrative scale which are identified by a location code. The location code directory of the Indian Census is formulated as per the recommendations of the Metadata and Data Standards Committee which adopts a new coding pattern for various administrative divisions which generally relates to governmental organizations (Office of the Registrar General & Census Commissioner, India, 2011). The scale of urban administrative units begins with *wards,* which are the lowest aggregation of urban areas local to *towns,* which are themselves grouped into *sub-districts,* further into *districts,* and finally into *states*, illustrated in Figure SI1.


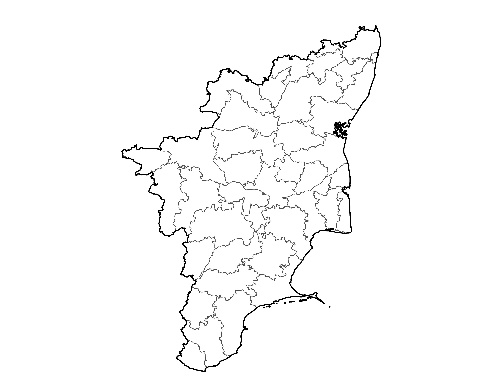

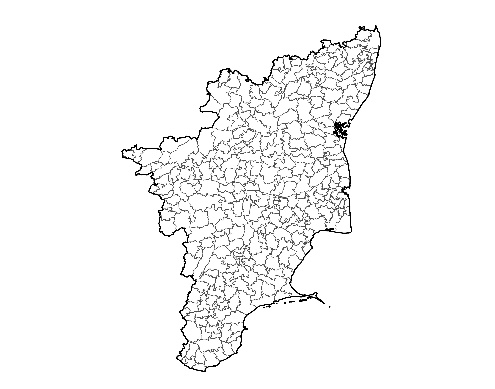

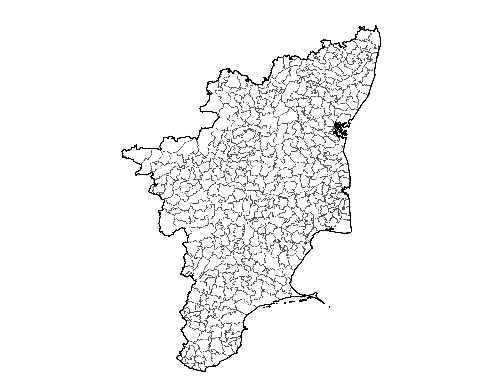

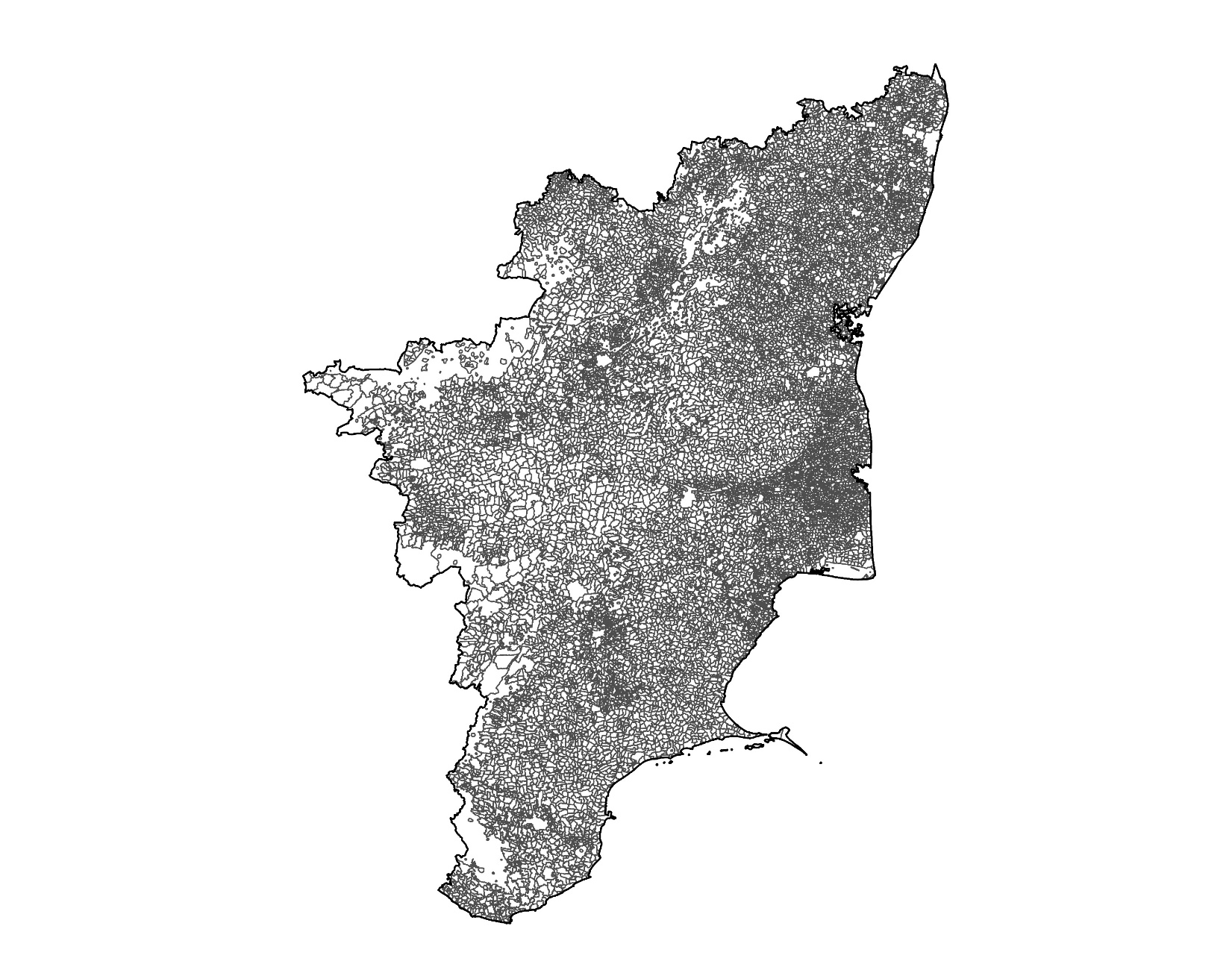


**A**

**B**

**C**

**D**

**Supplementary Figure 1:** **Geographies of**  **analysis.** Indicative diagram of the administrative scales of (A) wards, (B) urban local bodies, ie, towns and cities, (C) sub-districts, and (D) districts, for the state of Tamil Nadu. Note that the boundary of the figures indicates the state boundary for Tamil Nadu. The evaluation of scale effects follows the aggregation of wards, ie, the administrative units within figure A, to the various administrative scales from B-D.

Towns are defined either as statutory towns, census towns or cities. Statutory towns relate to administrative units defined by a statute or local governing body such as a Municipal Corporation. Census towns are defined by the simultaneous achievement of three criteria:

1) a minimum population of 5,000 persons,

2) 75% or above of the male main working population engaged in non-agricultural pursuits, and

3) a population density of at least 400 persons/km^2^.

Cities are then classified as census towns with a population of 100,000 or above (Office of the Registrar General & Census Commissioner, India, 2011). We do not further disaggregate census towns by the varying definitions and therefore refer to the scale of towns as towns and cities.

# City definition

The selection of cities is defined as those containing at least 30 local administrative divisions, ie, wards. This value is used as a rule of thumb to increase the confidence interval of the data when calculating the mean and standard deviation leading to the average heterogeneity index. However, in doing so, the city sample size is reduced significantly, from 4027 towns and cities to 524. As a result, the number of states included is reduced from 33 to 24. As such, the following analysis estimates the sensitivity of the results to this rule of thumb, reducing the number of wards required to define a city from 30 to 15 in increments of 5.

The results are presented in Table SI2 and indicate that, as more cities are included due to the decreasing requirement for the number of wards, the scale dependence of the heterogeneity measure tends to increase slightly as well as the estimated magnitude of heterogeneity, ie, the $b$ value increases marginally but with overlapping confidence intervals compared to other definitions, in some cases such that there is no statistically observable change. The most significant observable changes in average heterogeneity are found for sub-districts and districts, however these changes remain marginal. The same results are observed for including cities which contain 25 wards as with including those with 30 wards. For the 20-ward definition, the results reveal that states are statistically distinguishable as with previous results. Towns and cities remain statistically indistinguishable from sub-districts by this definition. Larger cities tend to have significantly more local urban administrative areas than smaller cities, and these cities tend to be simultaneously under the administrative control of city, sub-district and district levels as defined by the census (2011). As such, the overlapping definition generally results in the average heterogeneity being statistically indistinguishable from each other across these scales.

The sensitivity analysis therefore reveals that increasing the number of smaller towns and cities within the assessment tends to increase the observed scale dependence of national heterogeneity characterized by the national basic needs profile. However, the average heterogeneity predicted at this scale is statistically indistinguishable across each definition given the overlapping confidence interval, which is also the case for multiple definitions at other scales.

**Supplementary Table 2: Heterogeneity Indices Sensitivity.** Sensitivity analysis of the average heterogeneity index estimation, $b$, to the city selection defined by the number of wards, or local urban administrative areas, within each city, ie, cities are included within the study if the number of wards is equal to or exceeds those stated in the table.

| Scale | No. of wards | Sample size | Heterogeneity index, b | 95% CI | Fit, r^2^ |
| --- | --- | --- | --- | --- | --- |
| Towns/cities | 30 | 524 | 0.324 | [0.303, 0.344] | 0.91 |
|  | 25 | 895 | 0.330 | [0.312, 0.348] | 0.91 |
|  | 20 | 1319 | 0.333 | [0.317, 0.349] | 0.91 |
|  | 15 | 2762 | 0.340 | [0.326, 0.354] | 0.91 |
| Sub-districts | 30 | 486 | 0.3345 | [0.312, 0.357] | 0.908 |
|  | 25 | 806 | 0.342 | [0.322, 0.361] | 0.91 |
|  | 20 | 1126 | 0.352 | [0.333, 0.370] | 0.931 |
|  | 15 | 1846 | 0.379 | [0.363, 0.396] | 0.913 |
| Districts | 30 | 321 | 0.357 | [0.333, 0.381] | 0.911 |
|  | 25 | 405 | 0.382 | [0.361, 0.403] | 0.928 |
|  | 20 | 462 | 0.411 | [0.391, 0.430] | 0.94 |
|  | 15 | 526 | 0.443 | [0.426, 0.461] | 0.946 |
| States | 30 | 24 | 0.440 | [0.407, 0.473] | 0.982 |
|  | 25 | 27 | 0.451 | [0.424, 0.477] | 0.989 |
|  | 20 | 29 | 0.462 | [0.434, 0.489] | 0.989 |
|  | 15 | 33 | 0.491 | [0.462, 0.520] | 0.988 |

## Overlapping administrative divisions

The results reveal minor changes to the average heterogeneity index at the scale of towns/cities when redefining towns/cities as those which are simultaneously towns/cities, subdistricts, and districts, Table SI3. In total, 320 towns/cities are omitted from the analysis and the average heterogeneity index changes from $b = 0.324$ to $b = 0.318$, values which are statistically indistinguishable from each other. Similarly, when assessing the average heterogeneity index for those towns/cities which are non-overlapping the average heterogeneity index changes from $b = 0.324$ to $b = 0.333$. While the overlapping administrative divisions are a driving factor for the scale independence at town/city, subdistrict, and district level, redefining towns/cities by their overlapping administrative divisions yields the same scale dependence between towns/cities and states, and only reduces the overall intra-urban heterogeneity marginally.

**Supplementary Table 3: Analysis of overlapping administrative divisions.** Results for the regression of σ($\bar{X}$) vs $\sqrt{\bar{X}(1- \bar{X})}$ at the town/city level for those towns/cities which are simultaneously towns/cities, subdistricts, and districts, ie, overlapping administrative divisions, in comparison to the original values.

| Towns/cities | No. of data points | Heterogeneity index, b | r^2^ | 95% CI |
| --- | --- | --- | --- | --- |
| Overlapping | 204 | 0.318 | 0.89 | [0.284, 0.352] |
| Non-overlapping | 319 | 0.333 | 0.93 | [0.318, 0.350] |
| Original | 524 | 0.324 | 0.91 | [0.304, 0.345] |


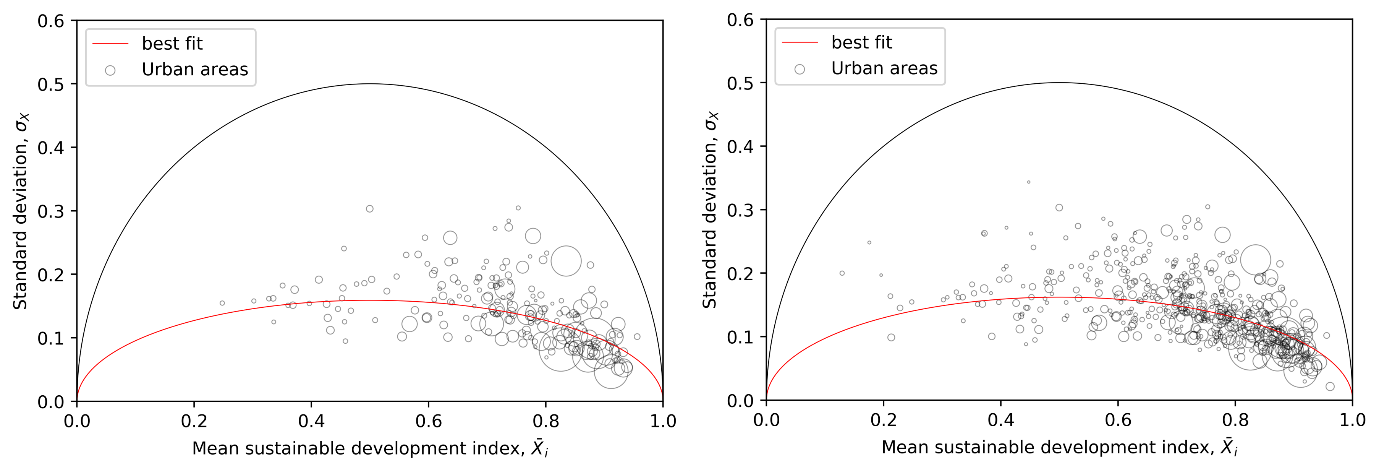


**Supplementary Figure 2: SDI Comparison of overlapping vs non-overlapping administrative divisions.** The relationship between the standard deviation of the SDI, $\sigma_{i}$, and the mean SDI, $\bar{X}_{i}$, at the town/city scale. (left) The relationship for those towns/cities which are simultaneously towns/cities, subdistricts, and districts, ie, overlapping administrative divisions, in comparison to (right) the relationship for those towns/cities which are non-overlapping. The profiles are calculated using SDI values for wards contained within each city. The size of the circle is proportional to the total urban population. The upper black line is the boundary of maximum heterogeneity, b = 1, and the lower black line, the x-axis, is the boundary of minimum heterogeneity, $b$ = 0. The red line represents the line of best fit calculated by regressing $\sigma_{i}$ on $\sqrt{\bar{X}_{i}- {\bar{X}_{i}}^{2}}$using population WLS regression.

# Fractional response model

Here, the dependent variable, ie, the SDI, is a measure of the rates of prevalence and is therefore bound within the range [0,1]. Therefore, due to the model specification of standard regression models, regression coefficients may yield fitted values which lie outside the upper and lower bounds of the SDI, ie, $SDI > 1$ or $SDI < 0$. Although such analysis still enables an indication of the direction of the perceived relationship, ie, positive or negative impacts, the magnitude of the effects may be highly inaccurate due to unrealistic predictions. Further, proportional data like that of the SDI is often distributed in an asymmetrical manner such that they may display heteroskedastic behavior and thus yield standard linear regression inappropriate (Bayer & Cribari-Neto, 2017).

Fractional response models concern outcomes bound within the range [0,1]. Such models assume values *within* the bounded range and therefore do not concern values equal to 0 or 1. However, this is appropriate here given that it would be unusual for urban areas to have a universal lack of access to at least one dimension, ie, $SDI = 0$, or universal access to all dimension, ie, $SDI = 1$. Specifically, beta regression is considered to model the behavior of average outcomes in terms of the prevalence of material stock compositions within urban areas.

## Beta regression

Beta regression is a relatively new fractional response model proposed by Ferrari and Cribari-Neto (Ferrari & Cribari-Neto, 2004). The general approach is to adopt a link function to map from the bounded space to a transformed space of ‘real numbers’. From here we perform a typical linear regression by maximizing the log-likelihood assuming the data follows a beta distribution.

Due to the versatility of the family of continuous probability distributions, or beta densities, bound between [0,1] as well as its ability to capture a range of uncertainties, scholars encourage its application to empirically understand a range of problems (Bayer & Cribari-Neto, 2017; Espinheira et al., 2019; Ferrari & Cribari-Neto, 2004). The beta regression model is formalized using the generalized linear model approach. To begin, the regression model is defined by assuming the data for the response variable, ie, the average basic needs outcomes measured by the SDI, is represented by the mean of a beta distribution $\mu_{t}$. This means that the beta regression can be modelled as presented in equation SI1.

$g\left( \mu_{t} \right)= \sum_{i=1}^{n} x_{ti}\beta_{i}$ [SI1]

Where $g$ is the link function, $\beta_{i}$ are the unknown regression parameters, $x$ are the independent variables ranging from $1$ to $n$, and $t$ relates to the data point. The mean of the beta distribution describing the SDI, $y$, is therefore transformed by the chosen link function. As we will see, the result of the model selection maximizing the log-likelihood and Bayesian Information Criteria (BIC) yield the complementary log-log as the most appropriate link function here, given in equation SI2. From here, the regression parameters, $\beta_{i}$, are estimated using a standard linear regression model by maximizing the log-likelihood. The model predictions are then interpreted by mapping back to the bounded space using the inverse link function, $g^{-1}$. However, before being able to conduct beta regression it is important to identify the appropriate link function and covariates which result in reliable predications of the perceived outcomes.

$g\left( \mu\right)=\ln(-\ln\left( 1-\mu\right))$ [SI2]

## Model specification

The model is specified by identifying the appropriate covariates and link function at the scale of towns and cities. The final model is then used for analysis at the remaining scales as well as to evaluate the relationship between the prevalence of built environment material stock compositions and specific societal services. To begin, the range of SDI values are evaluated to identify the population of towns and cities enabling regression analysis. This reveals a single census town which achieves an SDI value outside the range $0<SDI<1$, with an $SDI = 0$. The town is therefore omitted from the analysis and assumed to have minimal impact on the overall results given that it only contains a total of 69 households. From here the importance of variables to be included within the model selection is assessed by adopting two key approaches, ie, univariate analysis and the forward selection process. From here, the appropriate link function is evaluated resulting in the final beta regression model. Finally, the derivatives with which to interpret the magnitude of the relationship^[[1]](#footnote-1)^ are calculated. The derivatives report the percentage change in the response variable, ie, the SDI, for a unit increase in covariates, ie, the prevalence of the material types identified as significant. This is required due to the linear regression analysis taking place in a mathematically transformed space. We conduct model selection and beta regression within the Stata software using the inbuilt beta regression function to specify covariates and link functions.

## Variable processing

Often too many variables are included in instances where a large number of variables are available. The composition of residential buildings in the form of brick walls and concrete roofs are most prevalent, accounting for the composition of 41% of all households within towns and cities on average and 45% of the total urban household composition of India. The second most prevalent material combination for households is brick and metal for walls and roofs respectively and accounts for only 8% of the total number of households on average. There are 90 listed household types which experience varying co-linearity. Here, the study concerns the composition of built environment stocks and therefore including a significant number of variables which are rarely prevalent within the model selection itself may result in inaccurate variable selection. The number of covariates is reduced based on the average prevalence and by considering only those with a positive relationship with overall basic needs outcomes, ie, variables with a positive regression coefficient. Univariate beta regression is firstly performed on those variables accounting for at least 1% of the composition of households on average among towns and cities. These variables account for 14 out of the 90 available variables corresponding to over 86% of the total households within the urban areas of India. The forward selection process is then performed for variables with a positive and significant impact on basic needs outcomes. This involves sequentially including variables with the greatest impact on basic needs outcomes identified within the univariate model and including only those with a positive and significant impact in the multivariable regression. This results in two variables which are related to improved basic needs outcomes.

## Link function

The final covariates are then included within the multi-variable beta regression to select an appropriate link function by maximizing the log-likelihood and minimizing the BIC. The beta regression model is evaluated using four key link functions, namely: the log-log, complementary log-log, logit, and probit link functions which are widely used in generalized linear models. The complementary log-log link function is found to be most appropriate with the greatest log-likelihood and lowest BIC for the final variable selection at the scale of towns and cities and is used for models at other scales, with results of this process presented in Table SI4.

**Supplementary Table 4: Link function comparison.** Link function selection for multi-variable beta regression at the town and city level, adopting Brick_Conc and Conc_Conc as covariates. The results indicate that the complementary log-log link function is most suitable for the final beta regression, with the lowest Bayesian Information Criteria (BIC) and log likelihood.

| Link function | AIC | BIC | Log likelihood |
| --- | --- | --- | --- |
| Complementary log-log | -814 | -797 | 411 |
| Log-log | -810 | -792 | 409 |
| Logit | -811 | -793 | 410 |
| Probit | -812 | -795 | 410 |

# Extension of Paper Results

We firstly illustrate the relationship between the composition of all built environment stock and overall basic needs outcomes by evaluating the prevalence of the material types used for walls, roofs, and floors among households across the towns and cities of India. The results are presented in Figure SI3, SI4, and SI 5, respectively.

As shown in the main paper, areas with a high composition of brick in walls tend to have high basic needs outcomes, here we also show a negative correlation between the prevalence of mud and basic needs outcomes. Further, no areas exhibiting a high composition of mud, stone, wood, plastic, thatch/bamboo, or other materials in walls achieve high basic needs outcomes, ie, SDI > 0.9. A slight positive correlation is shown between the prevalence of concrete in roofs and basic needs outcomes and a negative correlation between handmade tiles and basic needs outcomes. Generally, we see that areas with a high achievement of basic needs outcomes, ie, SDI > 0.9, are associated with a high prevalence of concrete roofs, brick walls, and cement or tiled floors. There is a slight positive correlation between the prevalence of brick and basic needs outcomes. A high prevalence of mud floors indicates low levels of basic needs outcomes and reveals a negative association between the prevalence of mud floors and basic needs outcomes. This is further supported as there are no towns and cities with a high prevalence of mud as the predominant material for floors, ie, >90%, and where basic needs outcomes are higher than 50% on average, ie, 𝑆𝐷𝐼 > 0.5. A similar association is found for concrete and handmade tiles for roofs. However, 14 towns and cities are identified where floor material is predominantly cement, ie, >90%, and where basic needs outcomes are lower than 50% on average, ie, 𝑆𝐷𝐼 < 0.5. However, these correspond to areas with a significantly lower population than seen on average and similar trends for less substantial materials are not found, ie, less substantial materials still appear to be not associated with high basic needs outcomes.


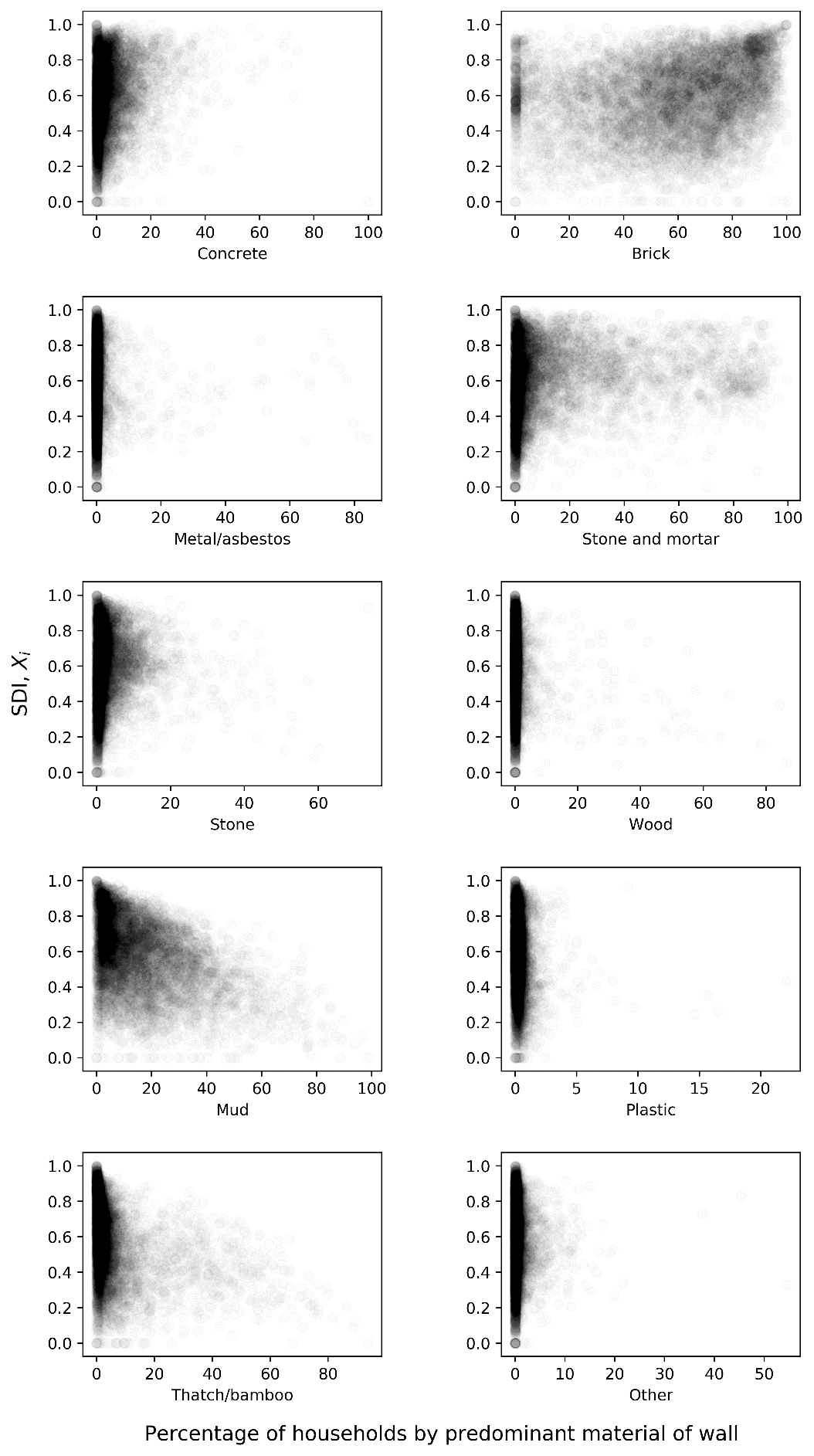


**Supplementary Figure 3: SDI versus predominate wall materials.** Average basic needs outcomes measured by the SDI versus the percentage of households present within the wards of India by predominant material of walls. Each data point corresponds to the proportion of households comprised of materials for the respective building element in a given area, thus the sum of the x-values for a given point in each panel results in 100%. See Figure B.2 for subplots for areas achieving SDI > 0.9.


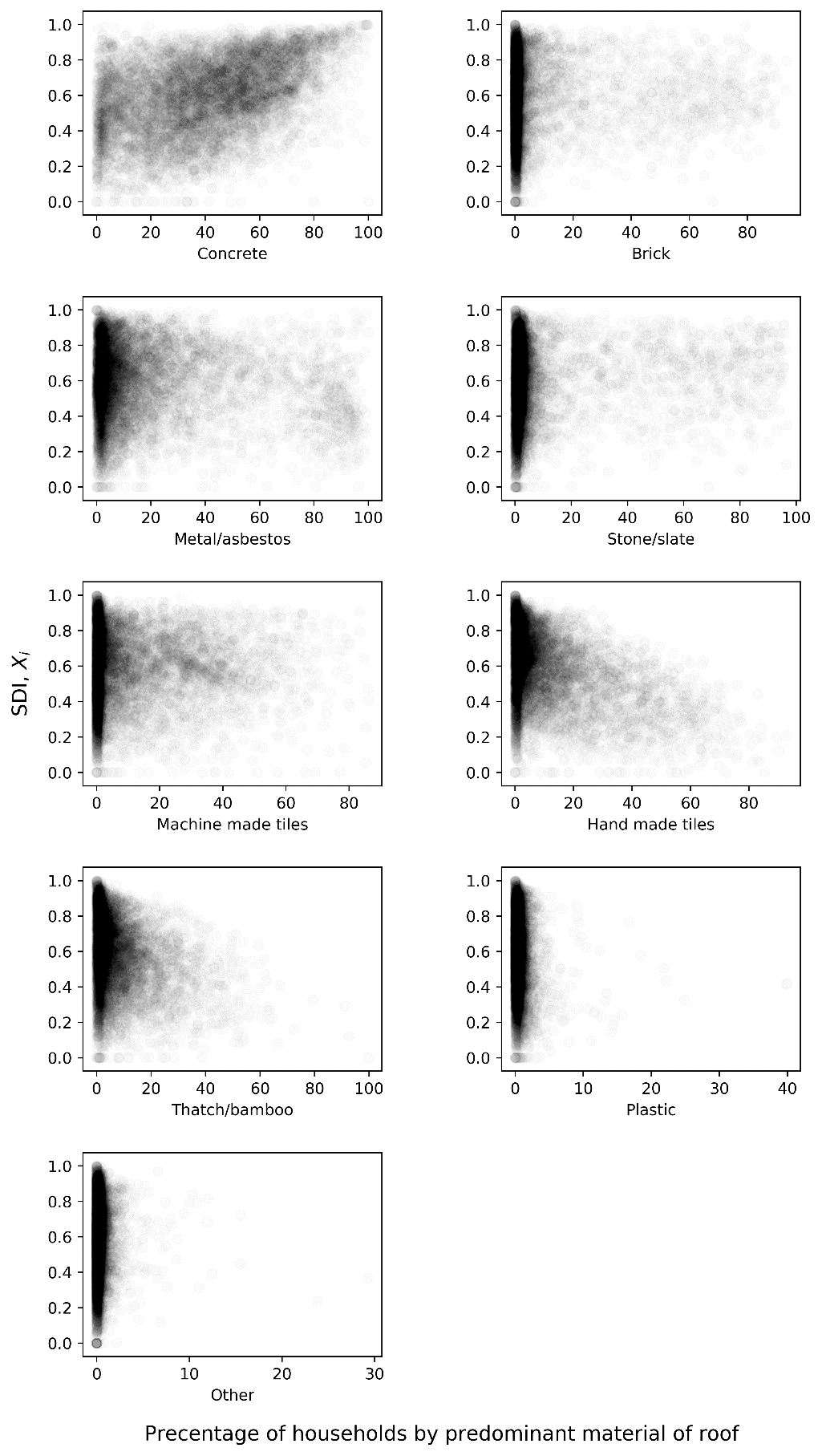


**Supplementary Figure 4: SDI versus predominate roof material.** Average basic needs outcomes measured by the SDI versus the percentage of households present within the wards of India by predominant material of roofs. Each data point corresponds to the proportion of households comprised of materials for the respective building element in a given area, thus the sum of the x-values for a given point in each panel results in 100%. See Figure B.3 for subplots for areas achieving SDI > 0.9.


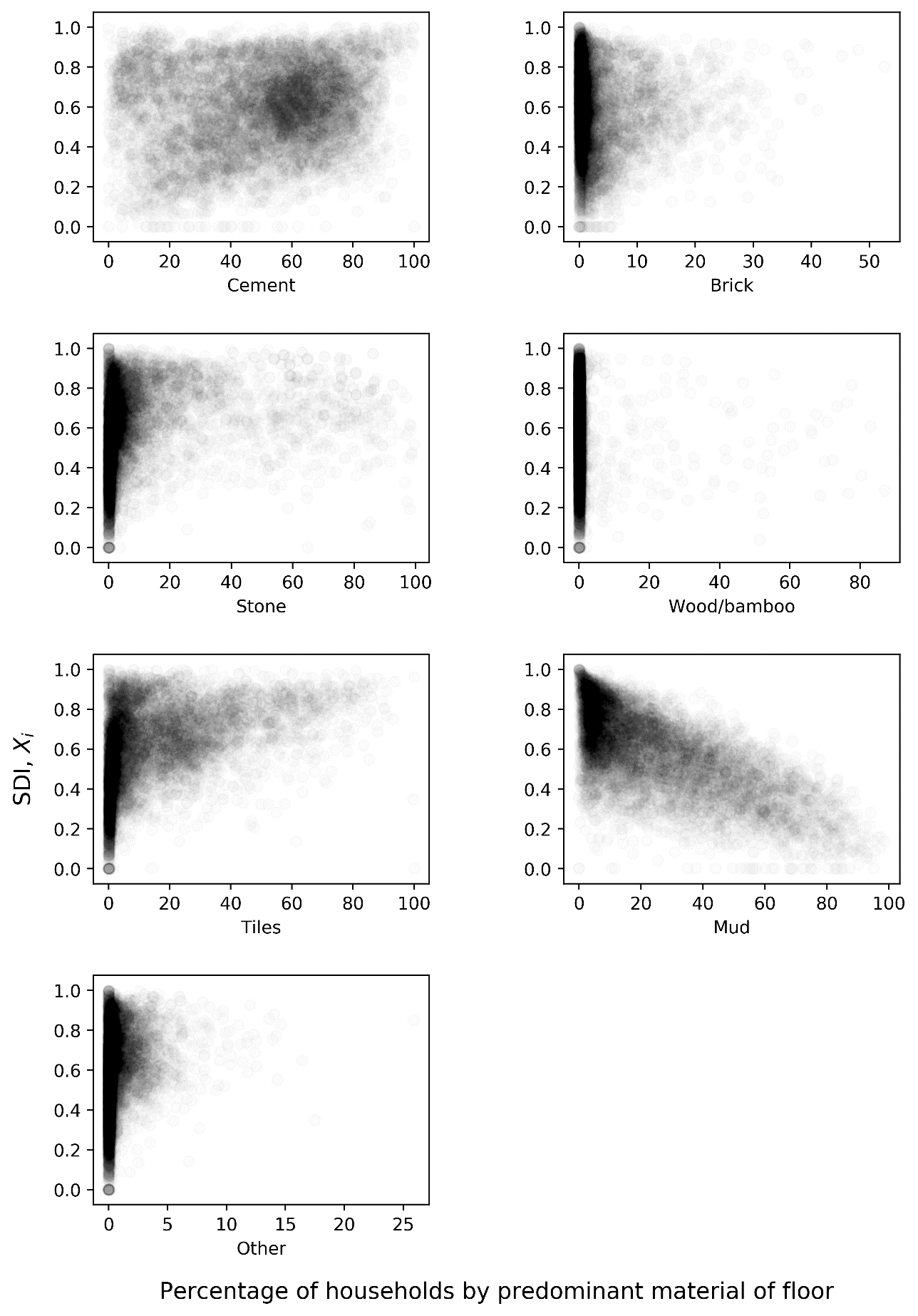


**Supplementary Figure 5: SDI versus predominant floor material.** Average basic needs outcomes measured by the SDI versus the percentage of households present within the wards of India by predominant material of floors. Each data point corresponds to the proportion of households comprised of materials for the respective building element in a given area, thus the sum of the x-values for a given point in each panel results in 100%. See Figure B.4 for subplots for areas achieving SDI > 0.9.

## Univariate analysis

**Supplementary Table 5:** **Univariate beta regression.** Results from regressing the listed variable, noted as material of *Wall_Roof* on the SDI. The mean composition refers to the average prevalence of households comprised of a unique combination of wall and roof materials among towns and cities. Note that stone for wall materials refers to stone packed with mortar. Bold variables indicate those with a statistically significant relationship with basic needs outcomes, using a $pvalue<0.05$.

| Variable | Mean composition (%) | P-value |
| --- | --- | --- |
| Brick_Conc | **41** | **0.00** |
| Brick_Metal | 8 | 0.34 |
| Brick_Stone/Slate | 7 | 0.36 |
| Brick_Brick | 5.7 | 0.31 |
| Stone_Conc | 5.7 | 0.09 |
| Conc_Conc | **4.4** | **0.00** |
| Brick_HandMadeTiles | **2.8** | **0.00*** |
| Brick_MachineMadeTiles | 2.4 | 0.64 |
| Mud_HandMadeTiles | 2.3 | 0.22 |
| Stone_Stone/Slate | **2.2** | **0.00*** |
| Mud_metal | **2.0** | **0.00*** |
| Mud_Thatch | **1.5** | **0.00*** |
| Stone_Metal | 1.3 | 0.57 |
| Total | **86.3%** | - |

*refers to variables which are found to have a significant but negative relationship with basic needs outcomes.

## Material composition of areas achieving high basic needs outcomes


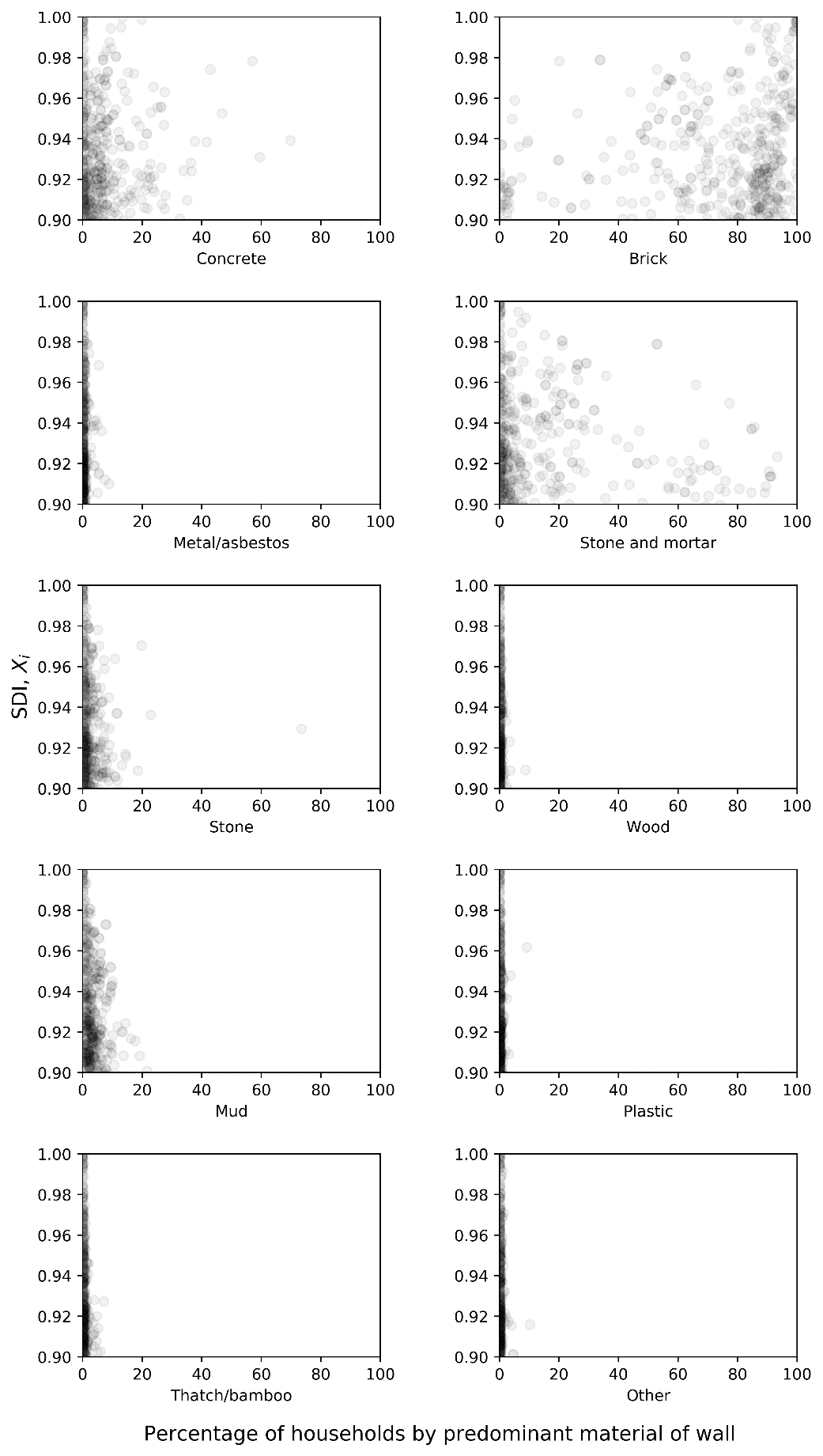


**Supplementary Figure 6: Wall material composition of areas achieving high basic needs outcomes.**  Average basic needs outcomes measured by the SDI versus the percentage of households present within the wards of India by predominant material of walls, for areas achieving an SDI > 0.9.


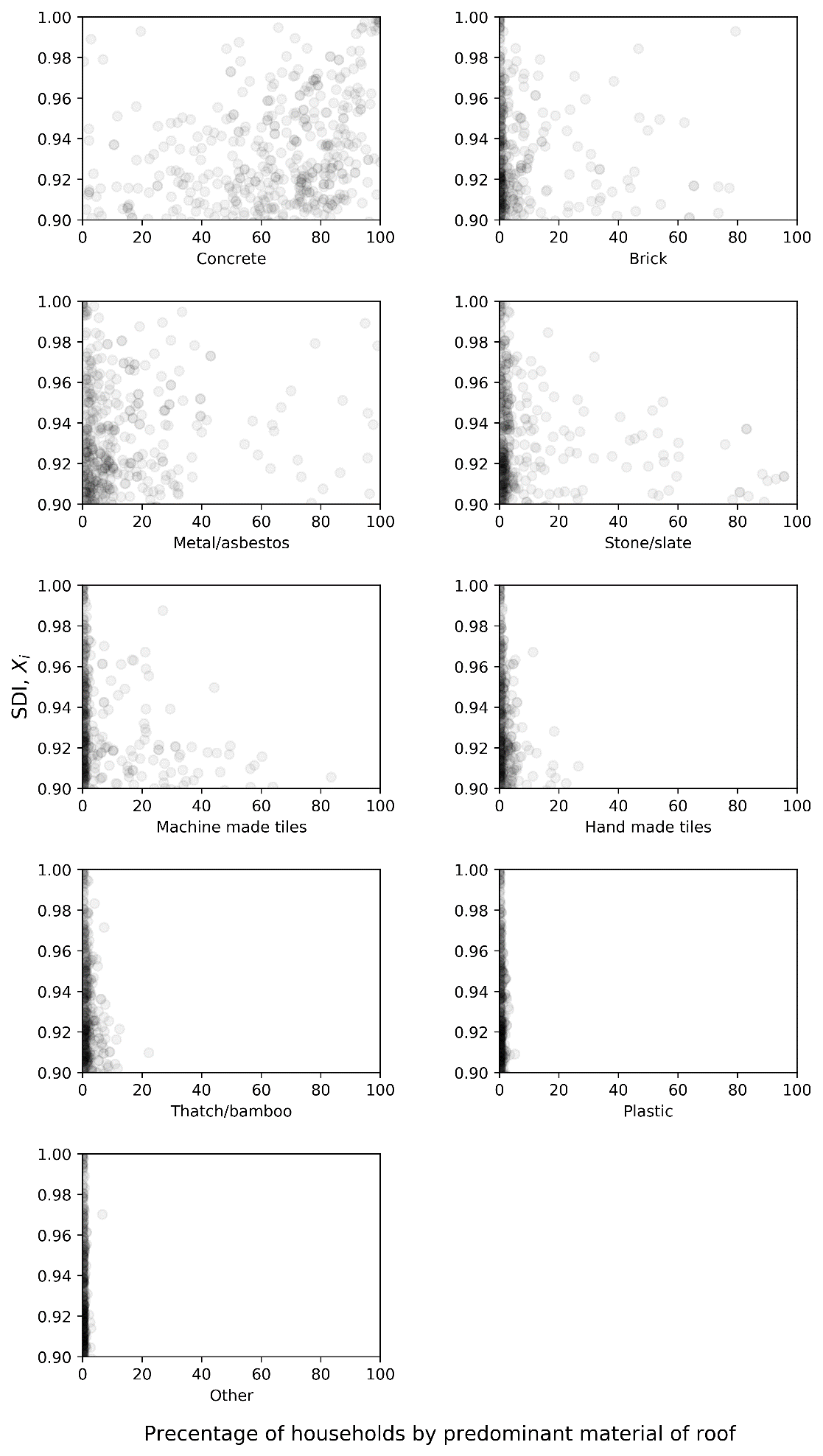


**Supplementary Figure 7: Roof material composition of areas achieving high basic needs outcomes** Average basic needs outcomes measured by the SDI versus the percentage of households present within the wards of India by predominant material of roofs, for areas achieving an SDI > 0.9.


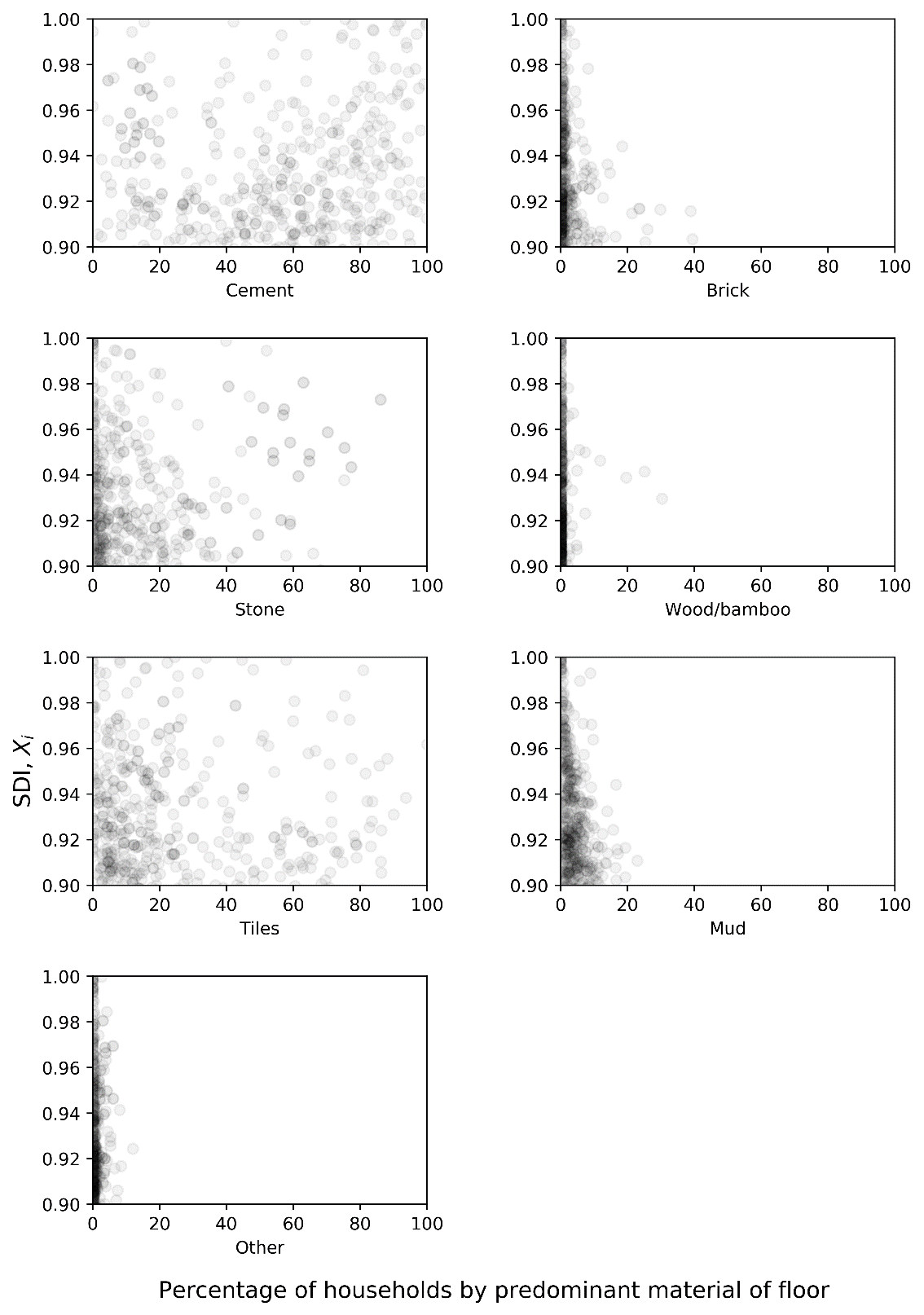


**Supplementary Figure 8: Floor material composition of areas achieving high basic needs outcomes.** Average basic needs outcomes measured by the SDI versus the percentage of households present within the wards of India by predominant material of floors, for areas achieving an SDI > 0.9.


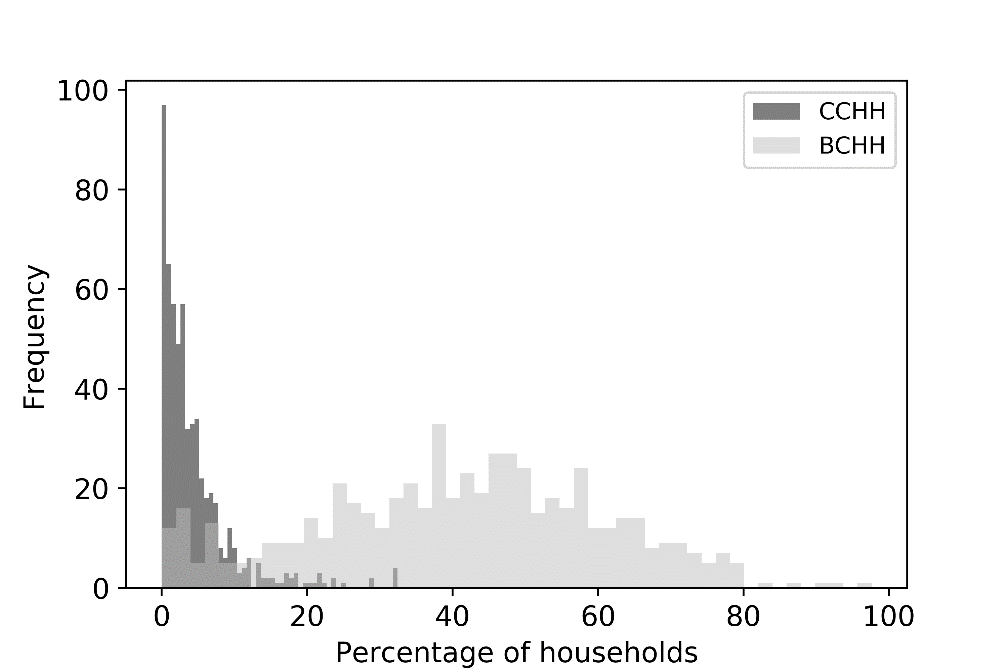


**Supplementary Figure 9:**  **Frequency of construction material.** The frequency distribution of Brick wall and Concrete roof household (CCHH) and Concrete wall and Concrete roof household (CCHH) across the towns and cities of urban India**.** The figure shows that BCHH is widely adopted and at a variety of rates, while CCHH is largely present in a small percentage of towns and cities and is mainly only responsible for a small percentage of the built environment in many areas.

# References

Bayer, F. M., & Cribari-Neto, F. (2017). Model selection criteria in beta regression with varying dispersion. *Communications in Statistics - Simulation and Computation*, *46*(1), 729–746. https://doi.org/10.1080/03610918.2014.977918

Broadhurst, D., Goodacre, R., Jones, A., Rowland, J., & Kell, D. (1997). Genetic alogrithms as a method for variable selection in multiple linear regression and partial least squares regression, with applications to pyrolysis mass spectrometry. *Analytica Chimica Acta*, *348*, 71–86.

Espinheira, P. L., Da Silva, L. C. M., Silva, A. D. O., & Ospina, R. (2019). Model Selection Criteria on Beta Regression for Machine Learning. *Machine Learning and Knowledge Extraction*, *1*(1), 427–449. https://doi.org/10.3390/make1010026

Ferrari, S., & Cribari-Neto, F. (2004). Beta Regression for Modelling Rates and Proportions. *Journal of Applied Statistics*, *31*(7), 799–815. https://doi.org/10.1080/0266476042000214501

National Institution for Transforming India. (2021). *Reforms in urban planning capacity in India*. Government of India. https://www.niti.gov.in/sites/default/files/2021-09/UrbanPlanningCapacity-in-India-16092021.pdf

Office of the Registrar General & Census Commissioner, India. (2011). *Census India*. https://censusindia.gov.in/2011census/hlo/HLO_Tables.html

1. More specifically, the derivatives refer to the average marginal effects. [↑](#footnote-ref-1)
